# Supplementary material for: PAX6 Downregulation Triggers HIF-1α-Mediated Ferroptosis in Glioma Cells
Source: Biomolecules. 2025 Oct 16;15(10):1462. doi: 10.3390/biom15101462 (PMC12564151; doi:10.3390/biom15101462)
Supplement: Supplementary file 1 [file biomolecules-15-01462-s001.zip › biomolecules-3906225-supplementary/biomolecules-3906225-supplementary figures.pdf]

Figure 1D PAX6

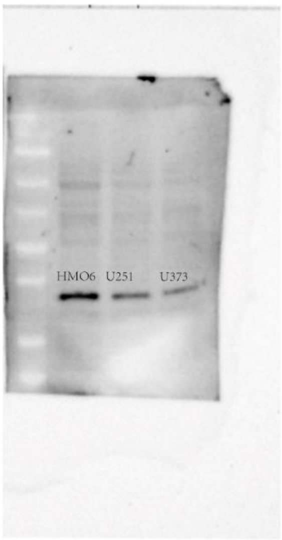

Figure 1D GAPDH

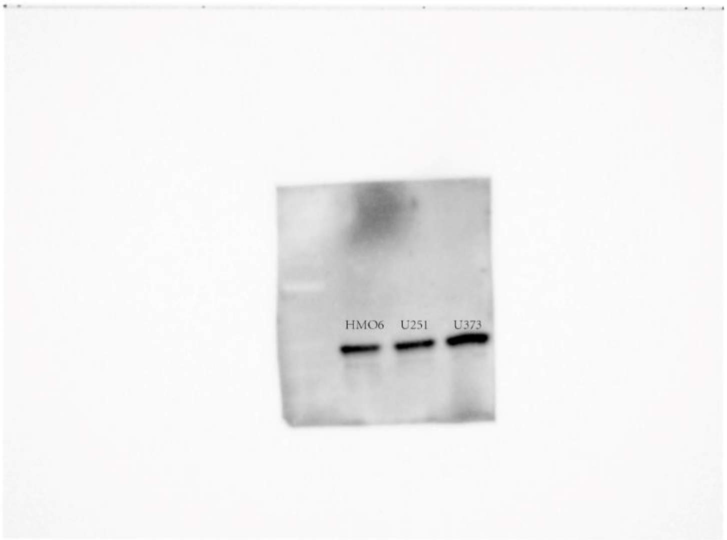

Note: To examine the protein expression level of PAX6, we performed Western blot analysis of total cellular proteins extracted from glioma cells (U251, U373) and normal glial cells (HMO6), with 30 µg of total protein loaded per well.

Figure 4E PAX6

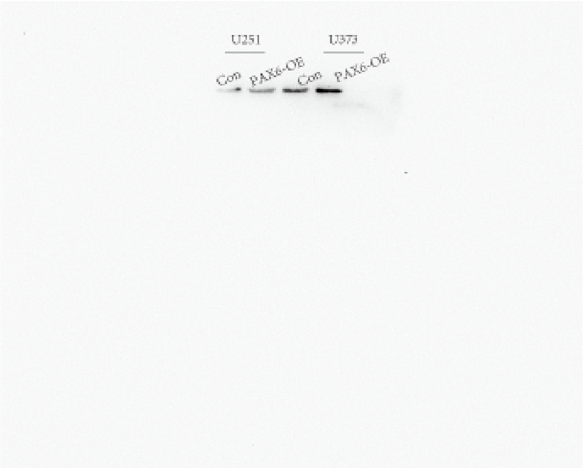

Figure 4E HIF1α

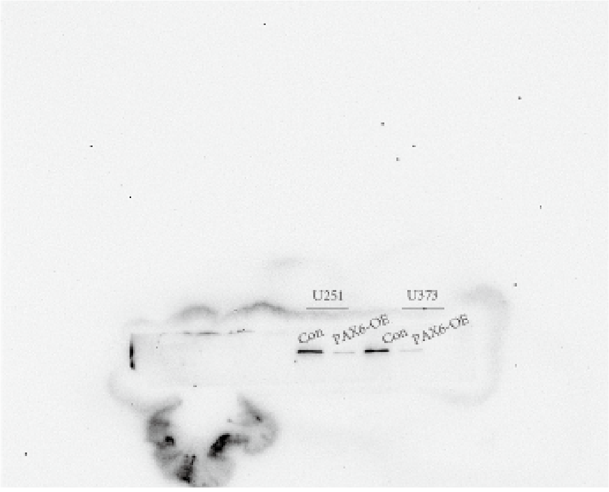

Figure 4E GAPDH

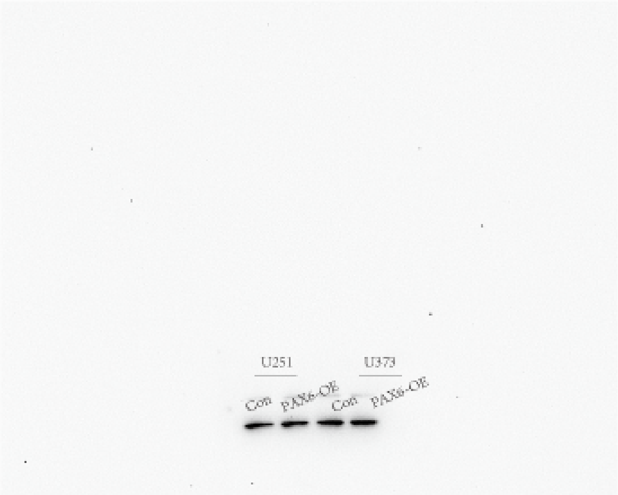

Note: To examine the protein expression level of HIF1α under PAX6 overexpression, total cellular proteins from glioma cells (U251, U373) were extracted and analyzed by Western blot, with 30 µg of total protein loaded per well.

Figure 4G HIF1α

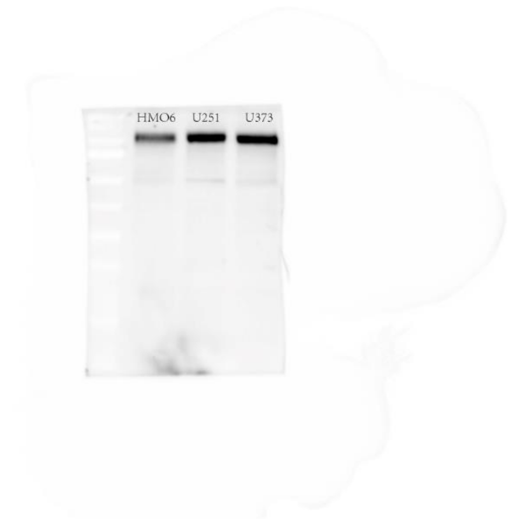

Figure 4G GAPDH

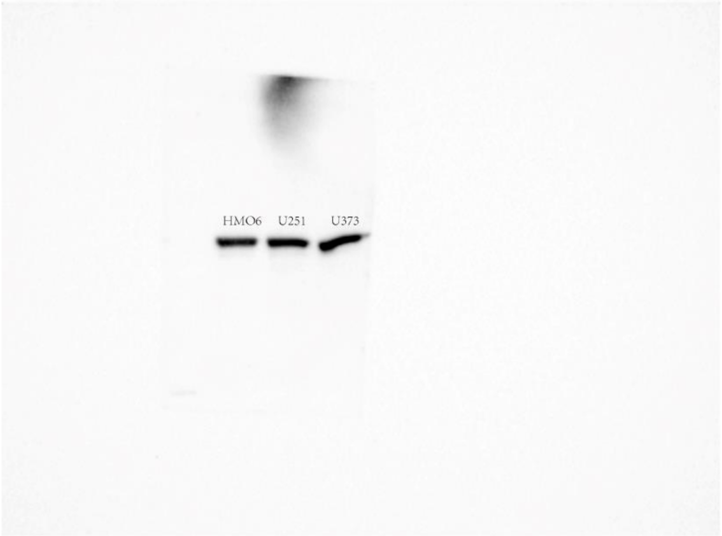

Note: To examine the protein expression level of HIF1α, we performed Western blot analysis of total cellular proteins extracted from glioma cells (U251, U373) and normal glial cells (HMO6), with 30 μg of total protein loaded per well.

Figure 5H GPX4

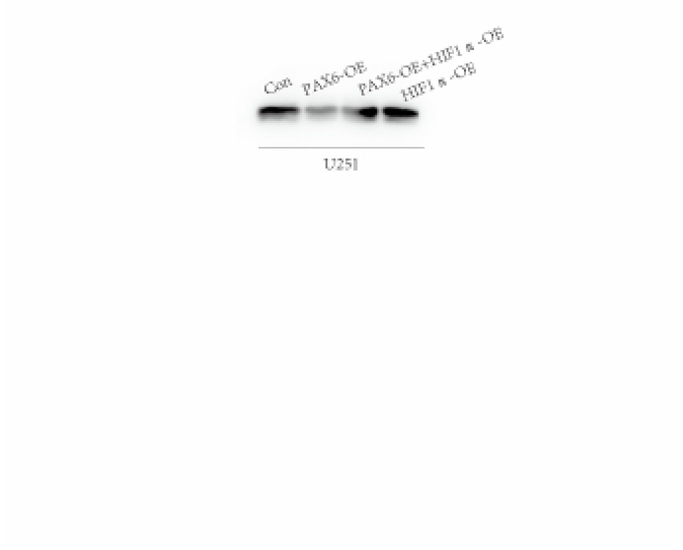

Figure 5H GAPDH

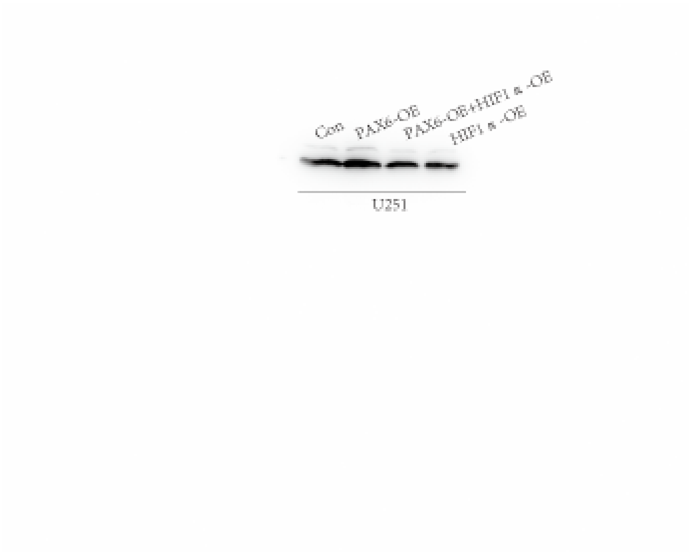

Note: To examine the protein expression level of GPX4 under different treatment conditions, total cellular proteins from glioma cells (U251) were extracted and analyzed by Western blot, with 30 μg of total protein loaded per well.

Figure 5I GPX4

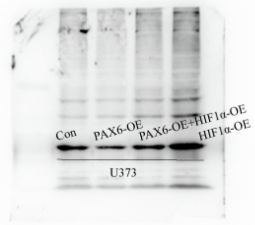

Figure 5I GAPDH

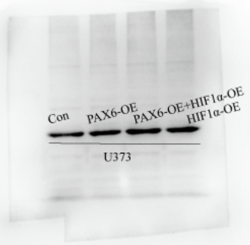

Note: To examine the protein expression level of GPX4 under different treatment conditions, total cellular proteins from glioma cells (U373) were extracted and analyzed by Western blot, with 30 μg of total protein loaded per well.

Figure 6E PAX6

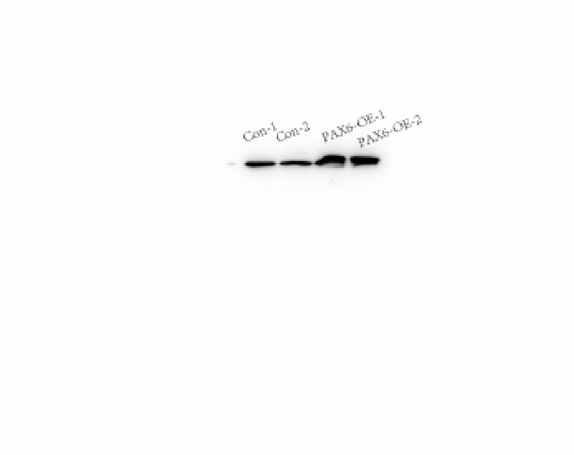

Figure 6E HIF1α

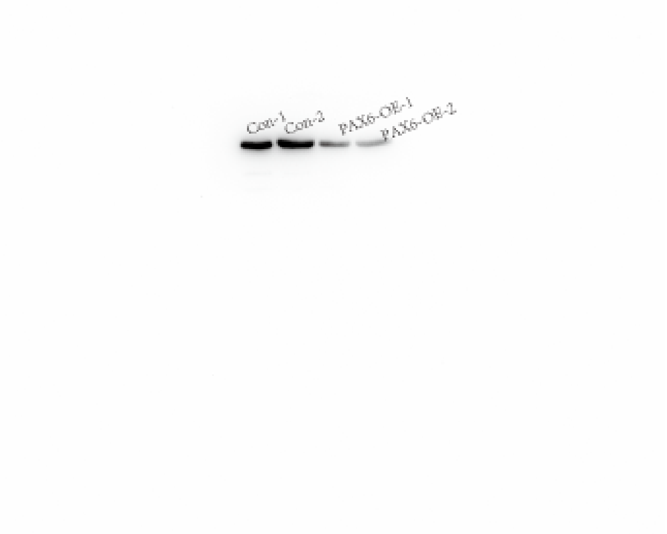

Figure 6E GAPDH

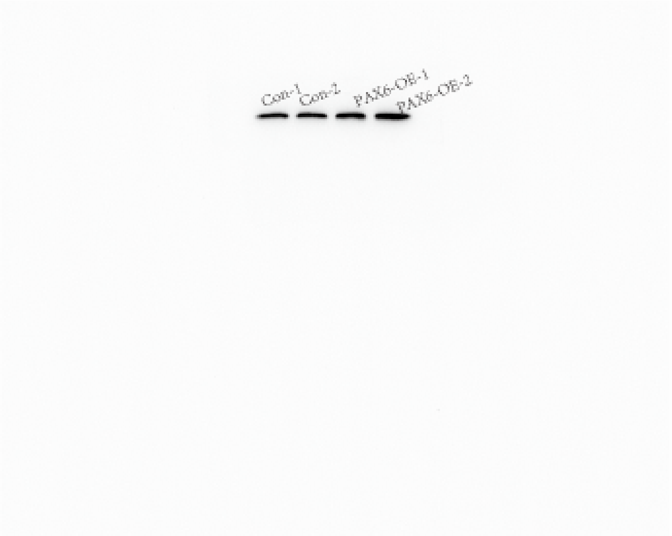

Note: To detect the expression levels of PAX6 and HIF1α in tumor tissues from the Control and PAX6-OE groups of nude mice, the tumor tissues were lysed, and total cellular proteins were analyzed by Western blot, with 30 μg of total protein loaded per well.

Figure 6F GPX4

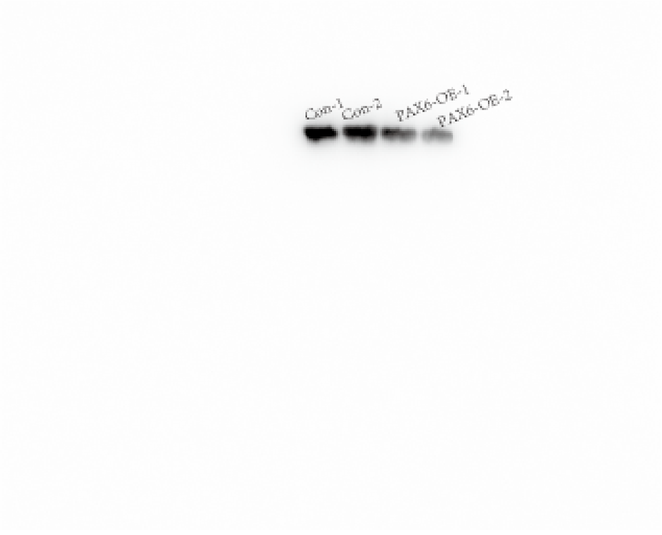

Figure 6F GAPDH

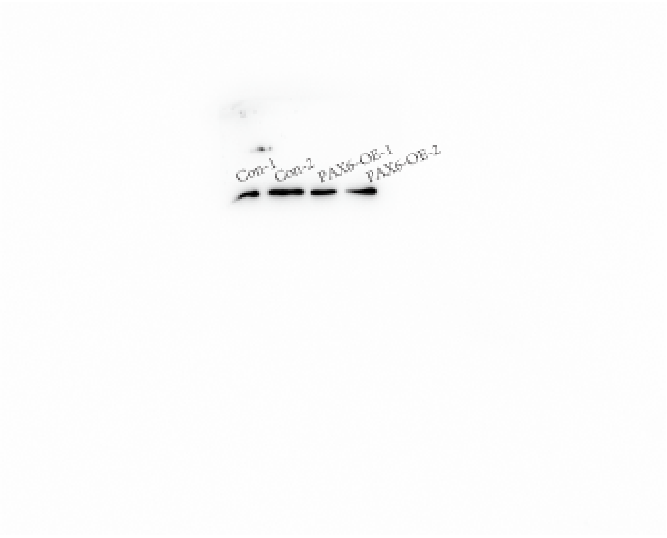

Note: To detect the expression levels of GPX4 in vivo from the Control and PAX6-OE groups of nude mice, the tumor tissues were lysed, and total cellular proteins were analyzed by Western blot, with 30 μg of total protein loaded per well.
