# Supplementary material for: PAX6 Downregulation Triggers HIF-1α-Mediated Ferroptosis in Glioma Cells
Source: Biomolecules. 2025 Oct 16;15(10):1462. doi: 10.3390/biom15101462 (PMC12564151; doi:10.3390/biom15101462)
Supplement: Supplementary file 1 [file biomolecules-15-01462-s001.zip › biomolecules-3906225-supplementary/biomolecules-3906225-supplementary tables.pdf]

Table S1| Spearman correlation analysis ρ value of PAX6_OE oxidative stress genes and iron metabolism related genes

Gene	*FTL*	*GPX4*	*HIF1AN*	*HIF3A*	*HMOX1*	*NFKB1*	*PAX6*	*SOD2*	*TFRC*	*TXNRD1*
FTL	1	-0.65714	-0.77143	0.880406	0.942857	0.771429	0.840668	0.657143	0.714286	0.6
GPX4	-0.65714	1	0.942857	-0.88041	-0.6	-0.77143	-0.57977	-0.82857	-0.88571	-0.94286
HIF1AN	-0.77143	0.942857	1	-0.88041	-0.65714	-0.82857	-0.57977	-0.77143	-0.94286	-0.82857
HIF3A	0.880406	-0.88041	-0.88041	1	0.880406	0.880406	0.770051	0.880406	0.758971	0.880406
HMOX1	0.942857	-0.6	-0.65714	0.880406	1	0.828571	0.927634	0.771429	0.6	0.657143
NFKB1	0.771429	-0.77143	-0.82857	0.880406	0.828571	1	0.753702	0.942857	0.771429	0.828571
PAX6	0.840668	-0.57977	-0.57977	0.770051	0.927634	0.753702	1	0.753702	0.637748	0.666737
SOD2	0.657143	-0.82857	-0.77143	0.880406	0.771429	0.942857	0.753702	1	0.714286	0.942857
TFRC	0.714286	-0.88571	-0.94286	0.758971	0.6	0.771429	0.637748	0.714286	1	0.771429
TXNRD1	0.6	-0.94286	-0.82857	0.880406	0.657143	0.828571	0.666737	0.942857	0.771429	1

Table S2| Spearman correlation analysis *P* value of PAX6_OE oxidative stress genes and iron metabolism related genes

Gene	***FTL***	***GPX4***	***HIF1AN***	***HIF3A***	***HMOX1***	***NFKB1***	***PAX6***	***SOD2***	***TFRC***	***TXNRD1***
FTL	NA	0.156175	0.072397	0.020599	0.004805	0.072397	0.036058	0.156175	0.110787	0.208
GPX4	0.156175	NA	0.004805	0.020599	0.208	0.072397	0.227784	0.041563	0.018845	0.004805
HIF1AN	0.072397	0.004805	NA	0.020599	0.156175	0.041563	0.227784	0.072397	0.004805	0.041563
HIF3A	0.020599	0.020599	0.020599	NA	0.020599	0.020599	0.073235	0.020599	0.080141	0.020599
HMOX1	0.004805	0.208	0.156175	0.020599	NA	0.041563	0.007666	0.072397	0.208	0.156175
NFKB1	0.072397	0.072397	0.041563	0.020599	0.041563	NA	0.083523	0.004805	0.072397	0.041563
PAX6	0.036058	0.227784	0.227784	0.073235	0.007666	0.083523	NA	0.083523	0.173071	0.14809
SOD2	0.156175	0.041563	0.072397	0.020599	0.072397	0.004805	0.083523	NA	0.110787	0.004805
TFRC	0.110787	0.018845	0.004805	0.080141	0.208	0.072397	0.173071	0.110787	NA	0.072397
TXNRD1	0.208	0.004805	0.041563	0.020599	0.156175	0.041563	0.14809	0.004805	0.072397	NA
